# Social Connectedness, Meals on Wheels Services and Healthcare Utilization Among High-Need Older Adults

**DOI:** 10.1093/geroni/igab046.3440

**Published:** 2021-12-17

**Authors:** Sarah Walsh, Jennifer Chubinski, France Weaver

**Affiliations:** 1 Eastern Michigan University, Ypsilanti, Michigan, United States; 2 Xavier University, Cincinnati, Ohio, United States

## Abstract

Homebound vulnerable adults 65+ are at an increased risk for social isolation and loneliness. The adverse consequences of loneliness are profound – including increased health care utilization, burden of dementia, chronic diseases, and mortality. Meals on Wheels (MOW) is a familiar source of nutritional support for homebound individuals who wish to stay in their homes and has additional important benefits. A growing body of evidence demonstrates that MOW provides mental and social health benefits beyond nutrition, but less is known about the interplay between MOW, social cohesion, and health services use. This project will address this gap in the literature using data from the 2013-2020 National Health and Aging Trends Study (NHATS), a nationally-representative panel study of 65+ Medicare enrollees. Using matching and longitudinal multivariate techniques, the risks of hospitalization and permanent nursing home entry are compared between MOW users and non-users. Our longitudinal dataset includes 11,266 observations. Of those, 12.8% rely on MOW or other food assistance (N= 1,488) and 16.6% experience low social cohesion (N= 1,936). Some 6.6% of participants are nursing home residents (N= 748) and the 39.1% report an overnight hospital stay in the prior year (N= 4,560). MOW is a comparatively low-cost intervention to help homebound older adults retain their independence and limit costlier healthcare utilization. This work extends our understanding of MOW services beyond simple nutrition benefits to its potential impact on social health.

